# The Processing of Visual and Phonological Configurations of Chinese One- and Two-Character Words in a Priming Task of Semantic Categorization

**DOI:** 10.3389/fpsyg.2015.01918

**Published:** 2016-01-05

**Authors:** Bosen Ma, Xiaoyun Wang, Degao Li

**Affiliations:** Department of Linguistics, School of International Studies, Zhejiang UniversityHangzhou, China

**Keywords:** Chinese words, visual configuration, phonological configuration, semantic representations, semantic categorization task

## Abstract

To separate the contribution of phonological from that of visual-orthographic information in the recognition of a Chinese word that is composed of one or two Chinese characters, we conducted two experiments in a priming task of semantic categorization (PTSC), in which length (one- or two-character words), relation, prime (related or unrelated prime-target pairs), and SOA (47, 87, or 187 ms) were manipulated. The prime was similar to the target in meaning or in visual configuration in Experiment A and in meaning or in pronunciation in Experiment B. The results indicate that the two-character words were similar to the one-character words but were less demanding of cognitive resources than the one-character words in the processing of phonological, visual-orthographic, and semantic information. The phonological primes had a facilitating effect at the SOA of 47 ms but an inhibitory effect at the SOA of 187 ms on the participants' reaction times; the visual-orthographic primes only had an inhibitory influence on the participants' reaction times at the SOA of 187 ms. The visual configuration of a Chinese word of one or two Chinese characters has its own contribution in helping retrieve the word's meanings; similarly, the phonological configuration of a one- or two-character word plays its own role in triggering activations of the word's semantic representations.

## Introduction

Most words that are orthographically similar are also similar to each other in pronunciation in an alphabetic language; it seems difficult to separate the processing of phonological from that of orthographic information in written word recognition. Written Chinese is of a logographic script. The orthographic units at the stroke, radical, and character levels and their combinations make Chinese visual orthography different from other languages (Chang et al., 2014, p. 289). A study of Chinese words appears helpful to reveal how the processing of phonological and visual-orthographic information contributes in initiating activations of representations for semantic information. We provide a new piece of evidence in this line of research in the present study.

### Chinese words

The basic units in written Chinese are Chinese characters. Most Chinese characters (e.g., 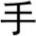 [shou3] *hand*) not only are words on their own but also can join with other characters to form two-character words (e.g., 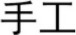 [shou3gong1] *handwork*) and words of more than two characters (e.g., 
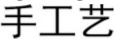
 [shou3gong1yi4] *handwork* and 
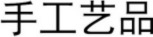
 [shou3gong1yi4pin2] *handicraft*). There are about three and a half thousand basic Chinese characters, and more than 70% of the 50,000 most frequently used words are two-character words (State language affairs committee, 2008).

#### The processing of a word's phonological information

A one-character word is often pronounced in the same way as are many other one-character words that are visual-orthographically different from it. For example, 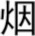 ([yan1] *smoke*) has 84 homophones (e.g., 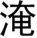 [yan1] *flood*), out of which 80 are dissimilar to it in visual orthography. Similarly, the pronunciation of the two-character word 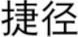 ([jie2jing4] *shortcut*) is the same as that of 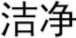 ([jie2jing4] *hygienic*), but these two words are not visual-orthographically or semantically related at all. Taking advantage of this similarity, researchers have proved that the processing of phonological information begins early in one-character word recognition (e.g., Perfetti and Tan, 1998; Weekes et al., 1998).

In a priming task of naming, for example, the participants' responses were facilitated to the targets (e.g., 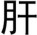 [gan1] *liver*) that were preceded by the homophone primes (e.g., 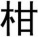 [gan1] *mandarin orange*) in comparison to their responses to those that were preceded by the unrelated primes (e.g., 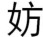 [fang2] *hamper*) at an SOA (stimulus onset asynchrony between the prime and the target in a trial) of 50 or 80 ms (ms) (Weekes et al., 1998). In a priming task of lexical decision, however, no phonological priming effect was obtained on a two-character target (e.g., 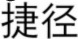 or 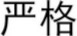 [yan2ge2] *strict*) that was preceded by its homophone (e.g., 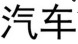) or by a pseudo-word (e.g., 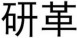 [yan2ge2]) (e.g., Zhou et al., 1999; Zhou and Marslen-Wilson, 2000, 2009; Wong et al., 2014).

Naming tends to reveal articulatory processes (Shen and Forster, 1999), and lexical decision is likely to involve a pattern match (Compton and Bradshaw, 1975). Meaning retrieval might not be necessary or be initiated by the processing of phonological information in these tasks (e.g., Taft and Van Graan, 1998). To address this problem, other tasks were adopted, such as semantic or phonological relatedness judgments (e.g., Perfetti and Zhang, 1995; Tan and Perfetti, 1999) and semantic categorizations (e.g., Chen et al., 1995; Leck et al., 1995; Sakuma et al., 1998). However, the results were not always consistent.

In semantic relatedness judgments, for example, the participants were reluctant to view the two sequentially presented homophonic one-character words (e.g., 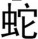 [she2] *snake*-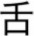 [she2] *tongue*) as semantically unrelated or to take the two semantically related words [e.g., 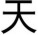 [tian1] *sky*- 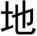 [di4] *earth*]) as phonologically unrelated (Perfetti and Zhang, 1995). The participants had longer response latencies and higher error rates when a two-character word (e.g., 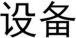 [she4bei4] *equipment*) was preceded by a homophone (e.g., 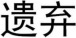 [yi2qi4] *abandon*) of its synonym (e.g., 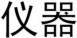 [yi2qi4] *instrument*) than by the control word (e.g.,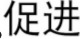 [cu4jin4] *promote*) (Tan and Perfetti, 1999).

In semantic categorizations, a homophone (e.g., *pair*) of an exemplar word (the word for a taxonomic category of basic level) (e.g., *pear*) was more difficult than the control word (e.g., *tail*) to reject as a member of the pre-designated category (e.g., *fruit*), because perception of the homophone caused activation of semantic representation for the exemplar word (Van Orden, 1987). The participants had to inhibit the activated representation for the exemplar word so as to make the correct judgment that the homophone target did not belong to the pre-designated category. Leck et al. (1995) indicated the importance of phonological information of Chinese compound characters in Van Orden's (1987) task of semantic categorization. Chinese characters can be divided into integrated and compound characters, but most Chinese characters are compound characters, each of which contains at least one radical that in most cases originates from an integrated character.

A similar study on Japanese two-kanji words yielded significant longer reaction times and higher error rates for a homophonic (e.g., 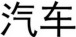 [kisya] *train*) than for a non-homophonic word (e.g., 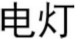 [dentou] *electric light*) of an exemplar word (e.g., 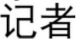 [kisya] *journalist*) (Sakuma et al., 1998). Also adopting Van Orden's (1987) task, however, Chen et al. (1995) failed to show a greater difficulty in categorizing a one-character homophone (e.g., 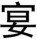 [yan4] *feast*) of an exemplar word (e.g., 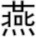 [yan4] *swallow*) as a member of the pre-designated category (e.g., 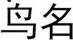 [niao3ming2] *bird*) than in doing the control word (e.g., 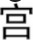 [gong1] *palace*).

#### The processing of a word's overall configuration

Many Chinese words (e.g., 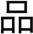 [pin3] *grade* and 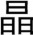 [jing1] *crystal*; 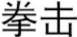 [quan2ji1] *boxing* and 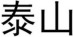 [tai4shan1] *Taishan Mount*) do not share components (radicals or characters) with but are similar to each other in visual configurations. One might wonder how the visual information of a one- or two-character word's overall shape is processed in the word's recognition.

In English, the representations for the words that are orthographically similar to each other form a neighborhood family. In a priming Stroop task, for example, the participants' naming of the color of the target (e.g., *bread*) was longer when the prime (e.g., *bead*) was visually similar than when it (e.g., *skill*) was unrelated to the target (Tanenhaus et al., 1980). Zhou et al. (1999) concluded that representations for a word's phonological, orthographic, and semantic information are closely associated. Given Tanenhaus et al.'s (1980) finding and Zhou et al.'s (1999) conclusion, one might deduce that the semantic representations for the one-character words that are similar to each other in visual configurations are closely associated. In Van Orden's (1987) task, for example, a one-character word (e.g. 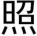 [zhao4] or 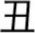 [chou3] *ugly*) that was similar in overall configuration to an exemplar word (e.g., 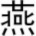 or 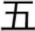 [wu3] *five*) of the pre-designated category (e.g., 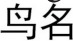 or 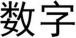 [shu4zi4] *number*) was more difficult than the control word (e.g., 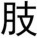 [zhi1] *limb* or 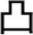 [tu1] *convex*) to reject inclusion into the category (Chen et al., 1995; Leck et al., 1995). The participants' perception of the distractor word that was similar to the exemplar word resulted in activations of semantic representations not only for the distractor word but also for the exemplar word. Competition between the activations caused interference in the participants' rejection of the distractor word.

Sakuma et al. (1998) adopted the same task as and appeared to achieve a similar result to Chen et al.'s (1995) on two-character words. Since the orthographic distractor (e.g., 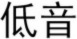 [di1yin1] *low-pitched sound*) shared a character (e.g., 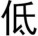 [di1] *low*) with the exemplar word (e.g., 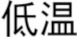 [di1wen1] *low temperature*), however, it might have been the character overlap between the two stimuli that had contributed to the observed effect of orthographic interference. To the authors' knowledge, unfortunately, no study seems to have been conducted on whether the perception of a two-character word (e.g., 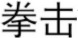) triggers activations of semantic representations for those (e.g., 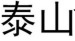) that are just similar to it in visual configuration.

Learners of written Chinese have to develop a knowledge of Chinese visual orthography (Siok and Fletcher, 2001; Wang et al., 2003). Compared to their visual processing of geometric figures, for example, children students' visual processing of Chinese characters' overall configurations is more closely related to character reading, indicating their development of visual-orthographic skills in the language (Luo et al., 2013). In the present study, we also took the words (e.g., 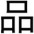 and 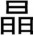; 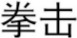 and 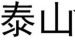) as similar in visual orthography that do not share components with but are similar to each other in visual configurations. It was expected that skilled readers' processing of both one- and two-character words' visual configurations was closely associated with their processing of the words' semantic information.

### The present study

Van Orden's (1987) task seems to have a strong point in that participants have to process the stimuli's phonological and orthographic information in an implicit way. As a matter of fact, Leck et al. (1995) and Sakuma et al. (1998) did seem to have successfully separated the processing of these two kinds of information, at least with one-character words as the stimuli. In a similar study, however, Chen et al. (1995) failed to find that the phonological distractor of the exemplar word resulted in longer reaction times than the control word.

Chen et al.'s (1995) failure to achieve their purpose probably indicated a weakness of Van Orden's (1987) task, in which participants make decisions on the exemplar-word target after reading aloud a preceding category name. In the sequence of two conscious processes there might be some freedom for participants to employ strategies. Thus, we speculated that a priming task of semantic categorization (PTSC) may be more sensitive than Van Orden's (1987) task in helping reveal how the processing of phonological and orthographic information parallels that of semantic information.

Van Orden's task appears susceptible to strategies whereas PTSC allows automatic semantic processing, but participants tend to perceive the primes' semantic information unconsciously in a priming task with a short SOA (e.g., 250 ms) (see Neely, 1991, for a review). Given associations between representations for a word's visual-orthographic, phonological, and semantic information (e.g., Zhou et al., 1999), participants' processing of the prime in a trial is expected to result in activations of semantic representations for a few words that are semantically, visual-orthographically, or phonologically similar to the prime in a PTSC. A semantic decision on a target will be facilitated if the prime is semantically, visual-orthographically, or phonologically similar to it.

Specifically, we wanted to show whether participants' living-or-non-living decisions on an exemplar-word target (e.g., 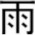 [yu3] *rain* or 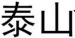) in a PTSC could be facilitated not only by the semantic (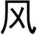 [feng1] *wind* or 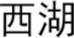 [xi1hu2] *West Lake*) but also by the visual-orthographic prime (e.g., 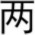 [liang3] *two* or 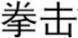) and whether their decisions on an exemplar word (e.g., 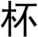 [bei1] *teacup* or 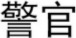 [jing3guan1] *policeman*) might be facilitated not only by the semantic (e.g., 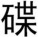 [die2] *dish* or 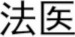 [fa3yi1] *court doctor*) but also by the phonological prime (e.g., 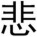 [bei1] *sad* or 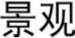 [jing3guan1] *landscape*). By living things we meant entities or parts of entities that belong to the categories of animals or plants; by non-living things we meant entities or parts of entities that belong to categories other than animals and plants. Thus, we arrived at the following hypothesis.

*If participants' perception of a one- or two-character word triggered activations of representations for a few words that are semantically, visual-orthographically, or phonologically similar to it, then their living-or-non-living decisions on an exemplar-word target in a PTSC would be influenced by the prime that was semantically, visual-orthographically, or phonologically similar to the target*.

We would also manipulate SOA (47, 87, and 187 ms) in order to make a time-course comparison of priming effect between one- and two-character stimuli. If similar patterns of priming effect would be achieved for one- and two-character stimuli at each SOA, then we would be able to provide a strong piece of evidence that two-character words can be both visual-orthographically and phonologically taken as wholes. We chose 47 ms as the shortest SOA because Wong et al. (2014) found a significant effect of facilitation with both the orthographic and the semantic primes of two characters at an SOA of 47 ms in a lexical decision task and Perfetti and Tan (1998) observed orthographic priming on one-character targets at an SOA of 43 ms in a naming task.

Since it is not common for an exemplar word to have both a word that is phonologically similar but not visual-orthographically or semantically related to it and a word that is visual-orthographically similar but not phonologically or semantically related to it at the same time, we conducted two experiments: Experiment A and Experiment B investigated how the processing of visual-orthographic and phonological information, respectively, contributed in activating representations for semantic information.

## Experiment A

### Method

The design formed a 2 (length: one- or two-character) × 2 (relation: visual-orthographic or semantic) × 2 (prime: prime or control) × 3 (SOA: 47, 87, or 187 ms) complex factorial with length, relation, and prime as three within-subjects variables and SOA as a between-subjects variable. The dependent variables were error rates and reaction times.

#### Participants

Fifty-four college students (37 females; *M*_age_ = 18.9 years, age range: 18.2–20.4 years) from engineering specialties were recruited on campus by means of a flier advertisement. They were divided into three groups randomly; each group would attend the experiment at one SOA. The present study was conducted according to the recommendations of the Ethics Committee of Zhejiang University. The participants gave written informed consent in accordance with the Declaration of Helsinki.

#### Materials

10 one- and 10 two-character exemplar words from 18 taxonomic categories were selected as the targets (see Appendix A in Supplementary Material). The semantic prime for each target was an exemplar word from the same category as the target but was visual-orthographically or phonologically unrelated to the target. To create 20 visual-orthographic primes for the targets, 25 students from the same pool as the participants wrote a response word to each target word on a piece of paper. The responses needed to be visual-orthographically similar to (having similar configurations to and not sharing radicals or characters with) but not semantically or phonologically related to the corresponding target stimuli. The response of the highest frequency to each target was taken as the visual-orthographic prime. (The responses whose frequency scores were lower than 25 percent were adopted for two reasons: First, they were also frequently taken as the corresponding alternatives in deaf and hard of hearing students' homework compositions, according to a Chinese teacher in Guangzhou Deaf School, China. Second, a discussion was conducted among a group of 9 graduate students majoring in linguistics, who were native speakers of Chinese, and an agreement was reached that they could be used as visual-orthographic primes in the present experiment.)

A third and a fourth group of 25 students evaluated the familiarity and the concreteness, respectively, of all the targets and the two types of primes. A fifth group of 25 students evaluated the typicality of the targets and the semantic primes. Since the visual-orthographic primes of one and two characters were different from the semantic primes of one, *t*_(9)_ = 4.753, *p* = 0.001, and two characters, *t*_(9)_ = 3.215, *p* = 0.011, in concreteness, respectively, we followed Zhou and Marslen-Wilson (2000) to create the control primes: For the semantic primes, 20 control words were determined that were not visual-orthographically, semantically, or phonologically related to the targets; similarly, 20 control words were determined for the visual-orthographic primes. The material raters did not participate in the experiment. Table 1 displays the sample stimuli and the property scores of the targets, the primes, and the controls at each treatment level of length by relation.

**Table 1:** **Sample stimuli and property scores in Experiment A**.

		**Sample**	Pinyin^*^	**Gloss**	**Frequency**	**Stroke**	**Familiarity**	**Concreteness**	**Typicality**
One-character	Target	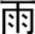	[yu3]	*Rain*	2.95	6.8	5.76	6.80	5.39
	VOP^**^	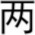	[liang3]	*Two*	2.97	6.2	4.47	4.45	–
	Control	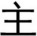	[zhu3]	*Main*	3.01	6.5	4.87	4.32	–
	SP	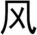	[feng1]	*Wind*	2.95	7.7	5.36	6.37	5.46
	Control	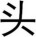	[tou2]	*Head*	2.87	8.8	5.23	6.50	–
Two-character	Target	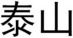	[tai4shan1]	*Taishan Mount*	1.60	13.0	4.83	6.69	5.11
	VOP	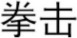	[quan2ji1]	*Boxing*	1.74	12.6	5.35	5.22	–
	Control	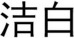	[jie2bai2]	*White*	1.78	13.7	5.32	5.28	–
	SP	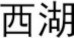	[xi1hu2]	*West Lake*	1.88	14.7	5.60	6.31	5.02
	Control	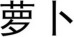	[luo2bo]	*Carrot*	1.93	13.6	5.61	6.50	–

* *Pinyin refers to the pronunciation system for Chinese characters*.

** *VOP, visual-orthographic prime; SP, semantic prime*.

*T*-test results indicated that the one- and the two-character semantic primes were not significantly different from the one- and the two-character targets, respectively, in word frequency, *t*_(9)_ = 1.042, *p* = 0.324; *t*_(9)_ = 1.447, *p* = 0.191, number of strokes, *t*_(9)_ = 0.714, *p* = 0.493; *t*_(9)_ = 1.182, *p* = 0.267, familiarity, *t*_(9)_ = 0.797, *p* = 0.446; *t*_(9)_ = 1.073, *p* = 0.311, concreteness, *t*_(9)_ = 1.721, *p* = 0.119; *t*_(9)_ = 1.456, *p* = 0.179, and typicality, *t*_(9)_ = 0.364, *p* = 0.724; *t*_(9)_ = 0.274, *p* = 0.790. The one- and the two-character visual-orthographic primes were not significantly different from the one- and the two-character targets, respectively, in word frequency, *t*_(9)_ = 0.261, *p* = 0.800; *t*_(9)_ = 1.548, *p* = 0.182, number of strokes, *t*_(9)_ = 1.500, *p* = 0.168; *t*_(9)_ = 0.480, *p* = 0.642, and familiarity, *t*_(9)_ = 1.833, *p* = 0.100; *t*_(9)_ = 0.854, *p* = 0.415. There were no significant difference between the semantic primes, the visual-orthographic primes, and the controls in word frequency, *F*_(1, 72)_ = 0.44, *p* = 0.508, number of strokes, *F*_(1, 72)_ = 0.18, *p* = 0.672, familiarity, *F*_(1, 72)_ = 0.09, *p* = 0.765, and concreteness, *F*_(1, 72)_ = 0.003, *p* = 0.860.

We created 144 one- and 144 two-character filler pairs in a similar way, and in each pair the prime was not related to the target at all. In a total of 368 trials, the percentage of related trials was 22%, and half the targets referred to living things. Then the trials were assigned to four blocks, which were not different from one another in numbers of critical trials, one-character trials, and trials with targets that referred to living things. Each critical target appeared once in a block, in which there were five critical trials at each treatment level of relation by prime. In two blocks, two and three of the five critical pairs of materials were of one and two characters, respectively, at each treatment level of relation by prime; in the other two blocks, three and two of the five critical pairs of materials were of one and two characters, respectively, at each treatment level of relation by prime.

The block order was counterbalanced across the participants, and the order of item presentations within a block was pseudo-randomized so that the targets of no more than four consecutively presented trials referred to living (non-living) things. Furthermore, the first and the last three trials of each block were fillers. The materials in each block were presented in the same order across the three SOAs, and the participants at each SOA would be required to respond to the same set of materials.

#### Procedure

We adopted DMDX (Forster and Forster, 2003) to control stimulus presentations and to record participants' responses. The experiment was conducted on personal computers. The screen resolution of the 15-inch screens was 1024 × 768 pixels. In each trial, a red fixation-cross “+” remained for 500 ms at the center of the white screen. Then a forward mask was presented for 200 ms. For the one-character primes, the mask (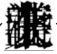) was constructed by superimposing eight different characters over a frame that was of the same size as one character and was referred to as a character mask. The mask for the two-character stimuli was simply a parallel presentation of two character masks. The prime stimulus was presented for 47, 87, or 187 ms at the center of the screen immediately after the mask disappeared. Then the target appeared and stayed at the center of the screen for 3000 ms or until a key press was received. Half the participants were required to press the key “Z” and the key “/” (and the other half the key “/”and the key “Z”) if the target referred to a living and a non-living thing, respectively. The two keys were horizontally separated by eight other keys. The inter-trial interval was 1000 ms. The stimuli were presented in the font “Song-36,” and the typeface was black. The first 16 trials were practice trials followed by the experimental trials. The participants each received 20 Yuan (3.3 USD) for their participation.

### Results

The data were deleted for the trials in which the reaction times were shorter than 200 ms or 3 *SD* above the overall average, and the ratio of the discarded data was 2.0%. Table 2 summarizes the results.

**Table 2:** **The participants' performance on the targets preceded by the primes and the controls at each treatment level of length by SOA by relation in Experiment A**.

			**Reaction time (ms)**				**Error rate (%)**			
			**Prime**		**Control**		**Prime**		**Control**	
			***M***	***SD***	***M***	***SD***	***M***	***SD***	***M***	***SD***
One-character	47 ms	VO^*^	696	86	702	63	4.6	6.6	1.1	3.2
		Semantic	699	85	689	80	2.3	4.4	3.3	5.9
	87 ms	VO	728	86	713	84	7.6	6.2	5.0	7.0
		Semantic	693	84	717	79	2.8	5.8	4.8	9.4
	187 ms	VO	732	74	697	71	6.1	8.8	6.0	7.2
		Semantic	686	94	714	70	4.3	6.3	4.8	8.0
Two-character	47 ms	VO	673	67	672	63	1.7	3.8	0.6	2.4
		Semantic	671	69	680	45	0.0	0.0	1.1	3.2
	87 ms	VO	684	72	681	61	2.4	4.6	1.7	3.8
		Semantic	665	91	693	60	1.7	3.8	2.8	4.6
	187 ms	VO	700	97	685	63	2.4	4.5	4.3	6.4
		Semantic	655	67	690	74	2.5	4.7	5.3	8.0

* *VO, visual-orthographic*.

#### Reaction times

**Figure 1:**
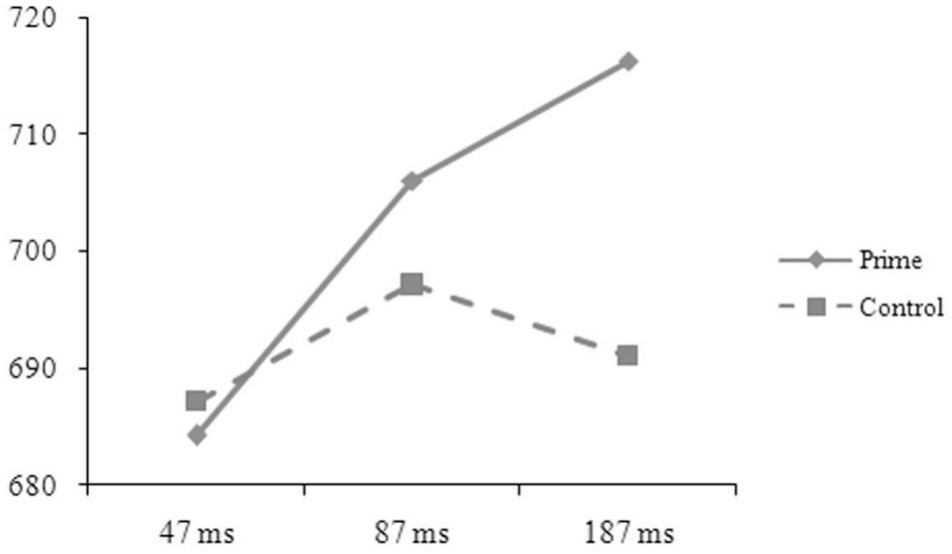
**The participants' reaction times to the targets preceded by the visual-orthographic primes and by the controls at different SOAs in Experiment A**.

**Figure 2:**
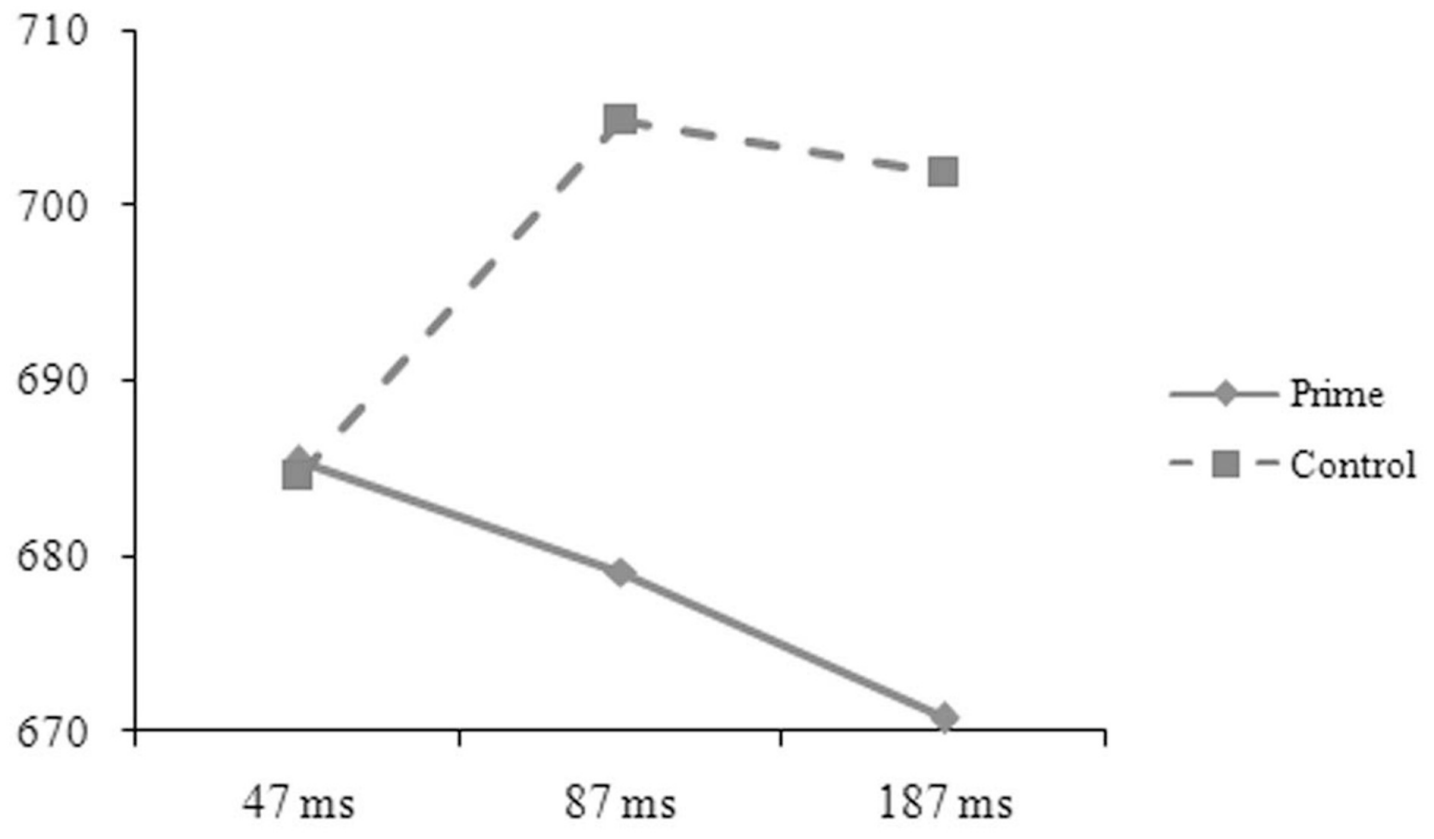
**The participants' reaction times to the targets preceded by the semantic primes and by the controls at different SOAs in Experiment A**.

2 (length: one- or two-character) × 2 (relation: visual-orthographic or semantic) × 2 (prime: prime or control) × 3 (SOA: 47, 87, or 187 ms) by-subject and by-item ANOVAs were conducted on the reaction-time data. The main effect was significant for length in the by-subject, *F*_1(1, 51)_ = 36.29, *p* < 0.001, $\eta_{p}^{2}=0.421$, but not in the by-item analysis, *F*_2(1, 72)_ = 1.63, *p* = 0.206, $\eta_{p}^{2}=0.022$. The participants' reaction times were significantly longer for the one-character (706, 10 ms) (*M, SE*) than for the two-character words (679, 8 ms). The two-way interaction was significant between relation and prime in the by-subject, *F*_1(1, 51)_ = 15.93, *p* < 0.001, $\eta_{p}^{2}=0.242$, but not in the by-item analysis, *F*_2(1, 72)_ = 0.38, *p* = 0.846, $\eta_{p}^{2}=0.001$. The three-way interaction was significant between relation, prime, and SOA (see Figures 1, 2), *F*_1(2, 50)_ = 5.78, *p* = 0.006, $\eta_{p}^{2}=0.188$; *F*_2(2, 144)_ = 3.42, *p* = 0.035, $\eta_{p}^{2}=0.079$. Simple effect analyses indicated that neither the visual-orthographic nor the semantic primes showed a significant priming effect at the SOA of 47 ms. At the SOA of 87 ms, the reaction times were significantly shorter for the targets preceded by the semantic primes (679 ± 81 ms) than by the controls (705 ± 66 ms), *t*_(17)_ = 2.499, *p* = 0.023, but were not significantly different for the targets preceded by the visual-orthographic primes (706 ± 72 ms) and the controls (697 ± 68 ms). At the SOA of 187 ms, the reaction times were significantly shorter for the targets preceded by the semantic primes (671 ± 67 ms) than by the controls (702 ± 64 ms), *t*_(17)_ = 3.301, *p* = 0.005, but were significantly longer for the targets preceded by the visual-orthographic primes (716 ± 80 ms) than by the controls (691 ± 55 ms), *t*_(17)_ = 2.240, *p* = 0.040. The other effects were not significant.

#### Error rates

**Figure 3:**
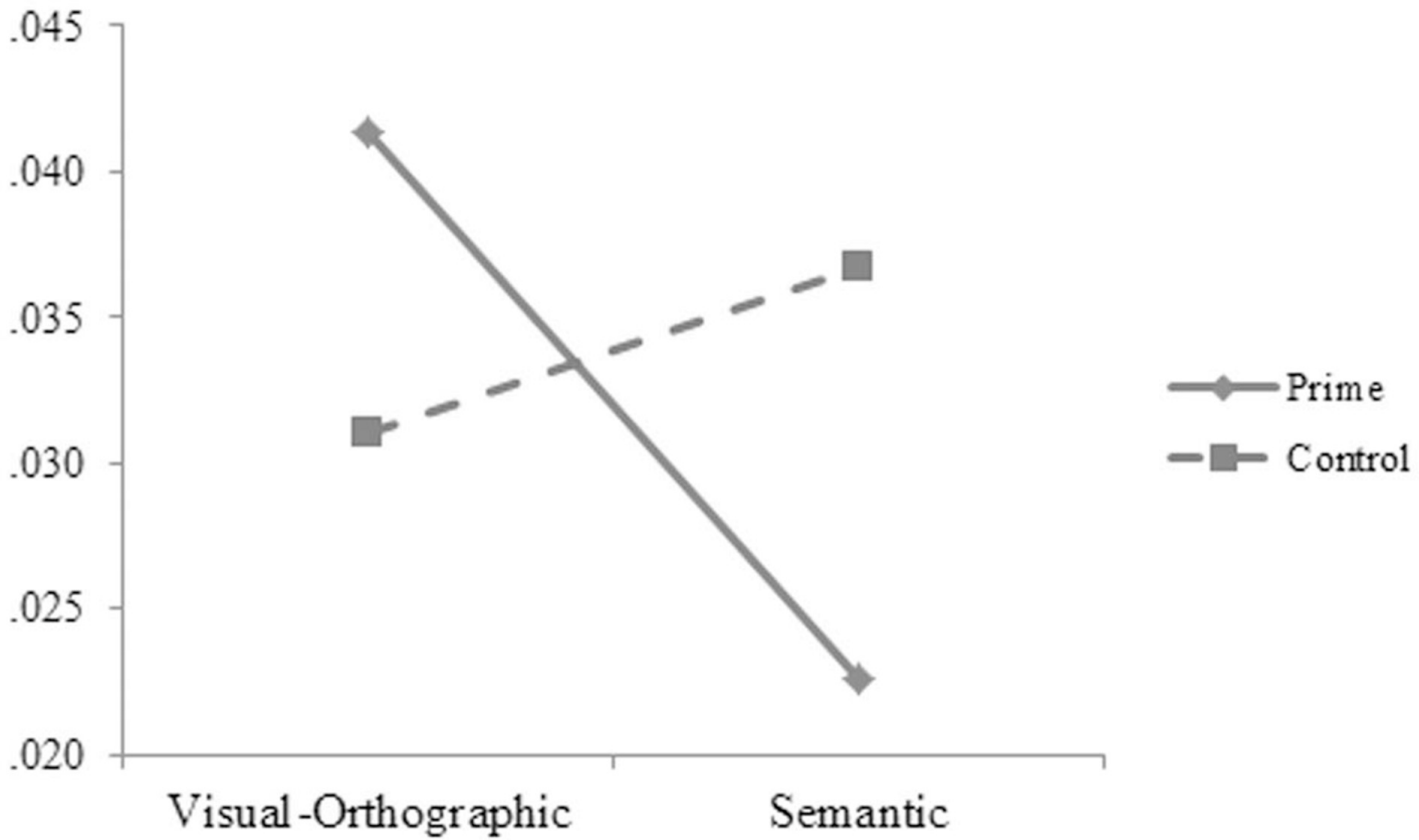
**The participants' error rates influenced by relation and prime in Experiment A**.

Similar analyses of the error-rate data showed that the main effect was significant for length, *F*_1(1, 51)_ = 16.43, *p* < 0.001, $\eta_{p}^{2}=0.247$; *F*_2(1, 72)_ = 6.84, *p* = 0.011, $\eta_{p}^{2}=0.087$. The error rates were significantly lower for the two- (2.2, 0.5%) than for the one-character stimuli (4.4, 0.7%). The main effect was significant for SOA in the by-item, *F*_2(2, 144)_ = 10.67, *p* < 0.001, $\eta_{p}^{2}=0.221$, but not in the by-subject analysis, *F*_1(1, 51)_ = 2.47, *p* = 0.095, $\eta_{p}^{2}=0.090$. The participants' error rates were significantly lower at the SOA of 47 ms (1.8 ± 3.3%) than at the SOA of 87 ms (3.5 ± 5.3%), *t*_(79)_ = 2.932, *p* = 0.004, and 187 ms (4.9 ± 6.2%), *t*_(79)_ = 4.464, *p* < 0.001. Their error rates were significantly lower at the SOA of 87 than 187 ms, *t*_(79)_ = 2.041, *p* = 0.045. The two-way interaction was significant between relation and prime (see Figure 3) in the by-subject, *F*_1(1, 51)_ = 6.44, *p* = 0.014, $\eta_{p}^{2}=0.114$, but not in the by-item analysis, *F*_2(1, 72)_ = 2.38, *p* = 0.129, $\eta_{p}^{2}=0.032$. Simple effect analyses suggested that the participants' error rates were significantly higher for targets preceded by the controls (3.7 ± 5.7%) than by the semantic primes (2.2 ± 3.6%), *t*_(53)_ = 2.384, *p* = 0.023. The other effects were not significant.

### Discussion

The participants had significantly shorter reaction times and lower error rates on the two- than on the one-character targets. Both the visual-orthographic and the semantic relations between the primes and the targets influenced the participants' responses, but the influences were independent of the stimuli's word length. These results will be discussed in General Discussion.

Furthermore, the semantic primes' facilitation became significant at the SOAs of 87 and 187 ms, in agreement with the prediction of the spreading activation model (SAM) (Collins and Loftus, 1975). That is, the participants' perceptions of the semantic primes resulted in activations of the semantic representations. The activations automatically spread to the targets' representations, resulting in a priming effect on the participants' reaction times.

The visual-orthographic primes, however, tended to inhibit the participants' responses in reaction times at the SOA of 87 ms, and the inhibition became significant at the SOA of 187 ms, similar to the finding in Dutch (Van Heuven et al., 2001), German (Drews and Zwitserlood, 1995), and English (Davis and Lupker, 2006; Nakayama et al., 2008) that mere orthographic similarity of word primes produced negative effects in a priming task of lexical decision. The inhibitive effect of the visual-orthographic primes on the participants' reaction times in the present experiment and in studies in alphabetic languages is in agreement with the prediction of the Interactive Activation Model (IAM) (McClelland and Rumelhart, 1981; Rumelhart and McClelland, 1982).

**Figure 4:**
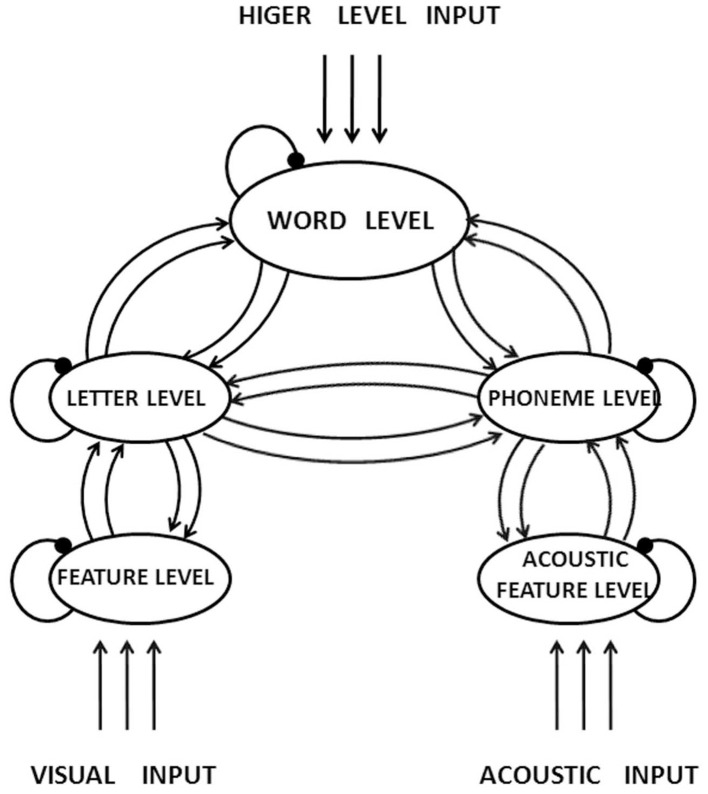
**The Interactive Activation Model (Rumelhart and McClelland, 1982)**.

The IAM assumes several levels of information processing in written word recognition (see Figure 4). Concerning the visual route of processing, information at the feature, the letter, and the word level is automatically processed in parallel. The inter-level communication can be both inhibitory and facilitating, whereas the intra-level connections are always inhibitory. Suppose the prime conveys orthographic information shared by the target in a priming task. At the presentation of the prime, entries for other words that are orthographically similar to the prime are activated and compete with each other to achieve a “survival of the fittest” outcome. Once the prime has exceeded a threshold level of activation, it starts to inhibit activations of representations for other words in competition, including the target, resulting in an inhibition of the target's activation.

Taft and Zhu (1997) proposed a modified version of the IAM and suggested that the subunits for a Chinese word are the strokes, the radical(s), the character(s), and the whole word. However, their model doesn't seem to presume the function of a word's overall configuration in activating its visual-orthographic neighbors (Yeh and Li, 2002). Since it must have been the similarity in visual configuration that resulted in the inhibiting effect for the visual-orthographic primes in the present experiment, we would add one more item to Taft and Zhu's (1997) list of subunits: a word's visual configuration at word level. It was the similarities between the primes and the targets in visual configurations at the word level that resulted in the inhibitory effect on the participants' responses.

Specifically, when the prime (e.g., 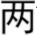) was presented, low spatial frequency information allowed a rapid extraction of the global configuration (Chua, 1999), resulting in activations of representations for the words (e.g., 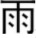, 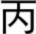 [bing3] *third*, and 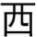 [xi1] *west*) that were similar to it in visual configuration. These candidates competed against one another for identification until the prime reached its threshold. At the very moment, the representations for those in competition were prevented from being further activated. In other words, the representation for the target (e.g., 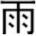) received an inhibition. By the time when the target was presented, cognitive resources were required to resolve the inhibition. Similar to the case of the prime, activation of representation for the target had to compete with those for the words that were similar to it in visual configuration. Meanwhile, the re-activation of the prime's representation in return made the target's recognition even more effortful.

The reason why facilitatory priming was observed in some previous studies on single characters (Feldman and Siok, 1999; Zhou and Marslen-Wilson, 1999) is because the visual-orthographic primes shared radicals with the targets. According to the IAM, inter-level communication happens in parallel with intra-level connections. In Feldman and Siok (1999) and Zhou and Marslen-Wilson (1999), for example, the information processing at the radical level at the early stage facilitated that at the character level, because similar radicals provide cues in both pronunciations and meanings. In the present experiment, the visual-orthographic primes were similar to the targets at the stroke level and in visual configurations. The similar strokes were not likely to result in shared radicals between the primes and the targets and might have contributed to the similarities of visual configurations at the character level. The participants' perceptions of the similarities between the primes and the targets as wholes could only result in intra-level connections, which were inhibitory as indicated by the IAM.

As the result of flow of information during word recognition with the early development of feed-forward inter-level facilitation and the later built-up intra-level inhibition (Ferrand and Grainger, 1992) the strength of inhibition increases (Drews and Zwitserlood, 1995), but there is sufficient time for a complete prime to ensure a conscious identification (Colombo, 1986; Segui and Grainger, 1990). This might be why error rates for the visual-orthographic targets increased as the SOA became longer but a reliable inhibition wasn't observed at the SOA of 187 ms in error rates.

These results are partially consistent with Perfetti and Tan (1998): graphic facilitation at the SOA of 43 ms gave way to inhibition at the SOAs of 57 and 85 ms in a naming task. However, the inhibitory effect in Perfetti and Tan (1998) seems to be earlier and larger than the one in the present experiment, probably because the members of an orthographic pair both had similar overall shapes and shared at least one radical with each other and were thus more confusable than the two members of a visual-orthographic pair in the present experiment. Similarly, Ferrand and Grainger (1992) found null effect for the orthographic primes at the SOA of 64 ms but a clear facilitory effect at the SOA of 32 ms. They believed that with the longer SOA, the between-level facilitation was canceled by the within-level inhibition.

Consistent with the findings that characters' visual configurations determine skilled readers' re-judgments of visual-orthographic similarity among characters (Yeh and Li, 2002) and that reaction times were significantly shorter in naming Chinese characters of symmetrical configurations than of non-symmetrical ones (Chen and Huang, 1999), the present experiment provides direct evidence not only on the processing of visual configuration in reading for meanings of one-character words but also on doing that of two-character words. Developing a global pattern instead of analysis of local details in word recognition (Yeh et al., 2003), skilled readers are sensitive to a word's visual configuration. It is even believed that the holistic processing is a mark of expertise for word perception, similar to expertise in face perception (Chen et al., 2013).

In summary, the finding that the visual configurations of one- and two-character words had an the inhibitory effect on the words' semantic processing may imply the following: Apart from the local features like strokes and radicals, the global shape of a word provides an independent source of information in activating visual-orthographically similar words, which compete with each other through mutually inhibitory connections.

## Experiment B

### Method

The design was similar to Experiment A except that a comparison of semantic and phonological priming effect was expected.

#### Participants

Fifty-four college students (34 females; *M*_age_ = 19.3 years, age range: 18.6–20.8 years) were recruited from the same pool and participated in the experiment in the same way as in Experiment A.

#### Materials

12 one- and 12 two-character exemplar words from 20 taxonomic categories (see Appendix B in Supplementary Material) were selected as the targets. The semantic primes were determined in the same way as in Experiment A. To create the phonological primes for the targets, 25 students from the same pool as the participants wrote a response word to each target word on a piece of paper that was phonologically similar to but was not semantically or visual-orthographically related to the target stimulus. The materials were evaluated in the same way as in Experiment A. Table 3 shows the sample stimuli and property scores. We created 136 one- and 136 two-character filler pairs. The percentage of related trials was 24%. The materials were assigned to four blocks in the same way as in Experiment A except that there were three one- and three two-character critical trials in each block at each treatment level of relation by prime.

**Table 3:** **Sample stimuli and property scores in Experiment B**.

		**Sample**	**Pinyin**	**Gloss**	**Frequency**	**Stroke**	**Familiarity**	**Concreteness**	**Typicality**
One-character	Target	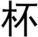	[bei1]	*Teacup*	2.85	8.8	6.11	6.42	5.26
	PP^*^	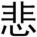	[bei1]	*Sad*	2.87	9.8	5.91	4.77	–
	Control	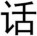	[hua4]	*Word*	3.01	9.7	5.97	4.83	–
	SP	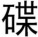	[die2]	*Dish*	2.78	8.5	6.28	6.59	5.45
	Control	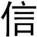	[xin4]	*Letter*	2.91	7.7	6.10	6.59	–
Two-character	Target	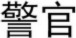	[jing3guan1]	*Policeman*	1.59	19.1	5.47	6.27	4.97
	PP	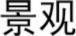	[jing3guan1]	*Landscape*	1.69	16.9	5.16	3.32	–
	Control	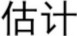	[gu1ji4]	*Estimate*	1.75	16.2	5.37	3.61	–
	SP	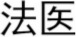	[fa3yi1]	*Court doctor*	1.93	17.5	5.91	6.43	5.05
	Control	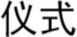	[yi2shi4]	*Ceremony*	1.87	17.3	5.71	6.35	–

* *PP, phonological prime; SP, semantic prime*.

The one- and the two-character semantic primes were not significantly different from the one- and the two-character targets, respectively, in word frequency, *t*_(11)_ = 0.898, *p* = 0.388; *t*_(11)_ = 1.479, *p* = 0.190, number of strokes, *t*_(11)_ = 0.279, *p* = 0.786; *t*_(11)_ = 0.584, *p* = 0.571, familiarity, *t*_(11)_ = 0.465, *p* = 0.651; *t*_(11)_ = 1.033, *p* = 0.324, concreteness, *t*_(11)_ = 0.883, *p* = 0.396; *t*_(11)_ = 0.946, *p* = 0.364, and typicality, *t*_(11)_ = 0.521, *p* = 0.613; *t*_(11)_ = 0.238, *p* = 0.816. The one- and the two-character phonological primes were not significantly different from the one- and the two-character targets, respectively, in word frequency, *t*_(11)_ = 1.060, *p* = 0.314; *t*_(11)_ = 1.226, *p* = 0.288, number of strokes, *t*_(11)_ = 0.673, *p* = 0.515; *t*_(11)_ = 1.122, *p* = 0.286, and familiarity, *t*_(11)_ = 0.611, *p* = 0.554; *t*_(11)_ = 0.590, *p* = 0.567, but not in concreteness, *t*_(11)_ = 3.248, *p* = 0.008; *t*_(11)_ = 9.816, *p* < 0.001. There were no significant difference between the semantic primes, the phonological primes, and the controls in word frequency, *F*_(1, 88)_ = 0.01, *p* = 0.919, number of strokes, *F*_(1, 88)_ = 0.22, *p* = 0.643, familiarity, *F*_(1, 88)_ = 0.02, *p* = 0.887, or concreteness, *F*_(1, 88)_ = 0.13, *p* = 0.717.

#### Procedure

The procedure was the same as in Experiment A.

### Results

One participant's data were excluded because he achieved an average error rate of over 30% at the SOA of 47 ms. Then the data were screened in the same way as in Experiment A, and the ratio of the discarded data was 2.2%. Table 4 displays the summary.

**Table 4:** **The participants' performance on the targets preceded by the primes and the controls at each treatment level of length by SOA by relation in Experiment B**.

			**Reaction time (ms)**				**Error rate (%)**			
			**Prime**		**Control**		**Prime**		**Control**	
			***M***	***SD***	***M***	***SD***	***M***	***SD***	***M***	***SD***
One-character	47 ms	Phonological	679	54	693	64	3.9	4.3	3.5	4.4
		Semantic	699	59	699	59	5.9	6.4	5.9	6.4
	87 ms	Phonological	704	59	696	59	6.6	6.2	3.4	5.3
		Semantic	684	72	710	66	5.1	5.1	6.6	7.9
	187 ms	Phonological	734	80	692	66	6.2	5.7	5.1	5.8
		Semantic	683	87	700	65	4.3	9.6	5.8	6.7
Two-character	47 ms	Phonological	673	53	690	52	3.0	5.1	2.0	3.7
		Semantic	680	68	686	46	0.0	0.0	1.0	2.8
	87 ms	Phonological	707	49	699	54	3.2	6.5	1.9	4.9
		Semantic	675	67	689	63	2.3	3.8	3.4	7.0
	187 ms	Phonological	699	78	689	74	1.5	4.6	0.9	3.9
		Semantic	660	75	712	64	0.5	2.0	1.5	3.4

#### Reaction times

**Figure 5:**
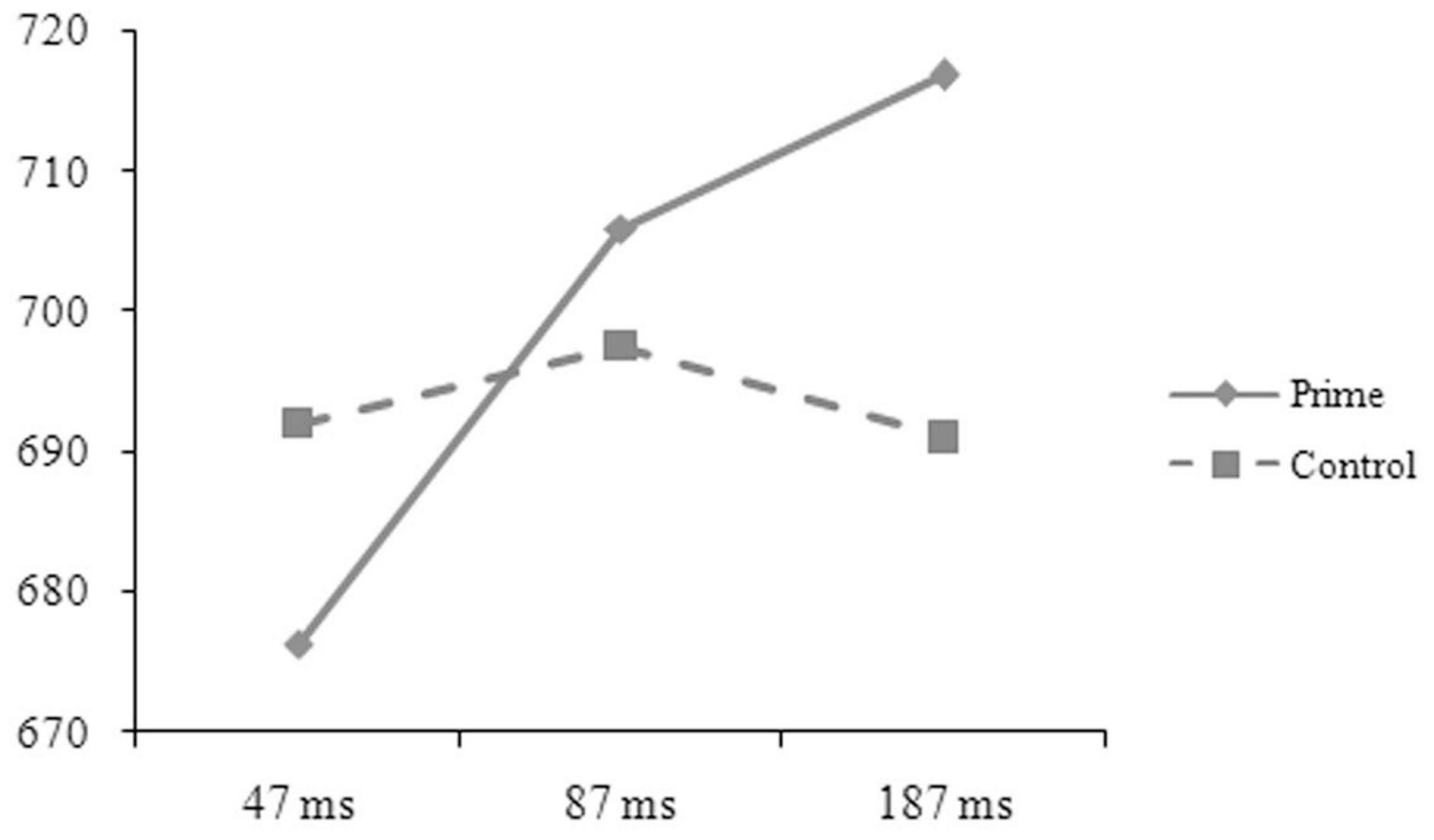
**The participants' reaction times to the targets preceded by the phonological primes and by the controls at different SOAs in Experiment B**.

**Figure 6:**
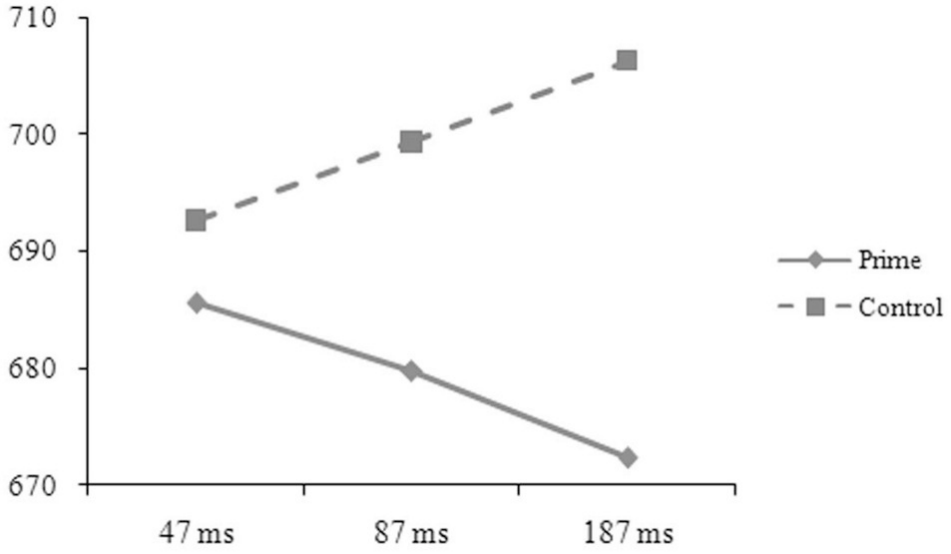
**The participants' reaction times to the targets preceded by the semantic primes and by the controls at different SOAs in Experiment B**.

The reaction-time data were analyzed in the same way as in Experiment A. The main effect was significant for length in the by-subject, *F*_1(1, 50)_ = 5.52, *p* = 0.027, $\eta_{p}^{2}=0.094$, but not in the by-item analysis, *F*_2(1, 88)_ = 0.49, *p* = 0.486, $\eta_{p}^{2}=0.006.$ The one-character targets (697, 8 ms) took longer time to respond to than the two-character targets (689, 7 ms). The main effect was significant for prime in the by-subject, *F*_1(1, 50)_ = 4.63, *p* = 0.036, $\eta_{p}^{2}=0.085$, but not in the by-item analysis, *F*_2(1, 88)_ = 0.21, *p* = 0.646, $\eta_{p}^{2}=0.002$, and was significant for SOA in the by-item, *F*_2(2, 176)_ = 3.98, *p* = 0.020, $\eta_{p}^{2}=0.086$, but not in the by-subject analysis, *F*_1(2, 50)_ = 0.19, *p* = 0.832, $\eta_{p}^{2}=0.007.$ The two-way interaction was significant between relation and prime in the by-subject, *F*_1(1, 50)_ = 101.37, *p* = 0.001, $\eta_{p}^{2}=0.185$, but not in the by-item analysis, *F*_2(1, 88)_ = 0.54, *p* = 0.466, $\eta_{p}^{2}=0.006.$ The three-way interaction was significant between relation, prime, and SOA (see Figures 5, 6), *F*_1(2, 50)_ = 6.33, *p* = 0.004, $\eta_{p}^{2}=0.202$; *F*_2(2, 176)_ = 5.57, *p* = 0.005, $\eta_{p}^{2}=0.118.$ Simple effect analyses indicated that the participants' reaction times were significantly shorter for the targets preceded by the phonological primes (676 ± 51 ms) than by the controls (692 ± 52 ms), *t*_(16)_ = 2.251, *p* = 0.039, at the SOA of 47 ms. No significant difference was found for the targets preceded by the semantic primes (686 ± 68 ms) and the controls (693 ± 44 ms), *t*_(16)_ = 0.767, *p* = 0.454. At the SOA of 87 ms, the participants' reaction times were significantly shorter for the targets preceded by the semantic primes (680 ± 65 ms) than by the controls (699 ± 52 ms), *t*_(17)_ = 2.358, *p* = 0.031, but were not significantly different for the targets preceded by the phonological primes (706 ± 48 ms) and the controls (697 ± 49 ms), *t*_(17)_ = 1.407, *p* = 0.177. At the SOA of 187 ms, their reaction times were significantly shorter for the targets preceded by the semantic primes (672 ± 72 ms) than by the controls (706 ± 56 ms), *t*_(17)_ = 3.371, *p* = 0.004, but were significantly longer for the targets preceded by the phonological primes (717 ± 70 ms) than by the controls (691 ± 65 ms), *t*_(17)_ = 2.314, *p* = 0.033. The other effects were not significant.

#### Error rates

**Figure 7:**
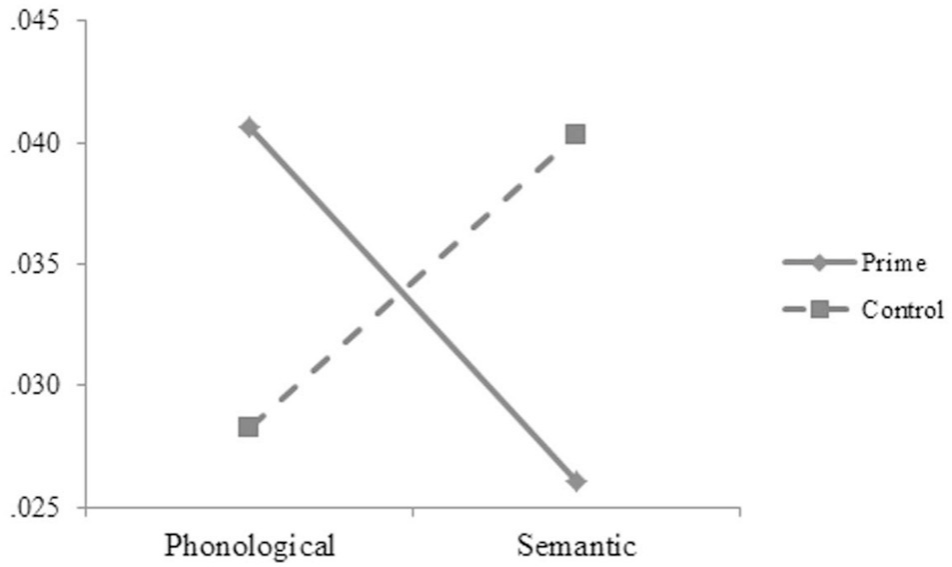
**The participants' error rates influenced by relation and prime in Experiment B**.

Similar analyses of the error-rate data showed that the main effect was significant for length, *F*_1(1, 50)_ = 54.19, *p* < 0.001, $\eta_{p}^{2}=0.520$; *F*_2(1, 88)_ = 4.60, *p* = 0.035, $\eta_{p}^{2}=0.050.$ The error rates were significantly lower for the two-character (1.8, 0.4%) than for the one-character stimuli (5.0, 0.6%). The main effect was marginally significant for SOA in the by-item, *F*_2(2, 176)_ = 2.46, *p* = 0.088, $\eta_{p}^{2}=0.027$, but not in the by-subject analysis, *F*_1(2, 50)_ = 0.66, *p* = 0.522, $\eta_{p}^{2}=0.026.$ The participants' error rates were significantly lower at the SOA of 47 ms (3.0% ± 8.1%) than at the SOA of 87 ms (4.2 ± 9.7%), *t*_(96)_ = 2.219, *p* = 0.029. The two-way interaction was significant between relation and prime (see Figure 7) in the by-subject, *F*_1(1, 50)_ = 7.36, *p* = 0.009, $\eta_{p}^{2}=0.128$, but not in the by-item analysis, *F*_2(1, 88)_ = 0.79, *p* = 0.376, $\eta_{p}^{2}=0.009.$ Simple effect analyses suggested that the participants' error rates were marginally higher for the targets preceded by the phonological primes (4.1 ± 4.0%) than by the controls (2.8 ± 3.8%), *t*_(52)_ = 1.927, *p* = 0.059, but were significantly lower for the targets preceded by the semantic primes (2.6 ± 4.0%) than by the controls (4.0 ± 4.9%), *t*_(52)_ = 2.163, *p* = 0.035. The other effects were not significant.

### Discussion

Similar to Experiment A, the participants performed better on the two-character than on the one-character targets, and their performance on these two types of stimuli was similarly influenced by the other three factors. At the SOA of 47 ms, the phonological primes significantly facilitated the participants' reaction times. This priming effect disappeared at the SOA of 87 ms when the semantic primes started to produce facilitation. At the SOA of 187 ms, a strong priming effect was observed for the semantic primes, but an obvious inhibition was observed for the phonological primes. The facilitation of the phonological primes at the shortest SOA suggests an early phonological processing, in agreement with Tan and Perfetti (1997, 1999). That phonological processing was weakened and semantic processing became dominant when SOA was extended to 87 ms confirms the argument that access to phonological information precedes semantic information (Perfetti and Tan, 1998).

The early and automatic phonological activation in word recognition seems to have its foundation. Students learn the form, the meaning, and the pre-determined pronunciation of a word at the same time. The integration of such practices is strengthened in their frequent contacts with the words in reading, which helps them gradually develop a strong awareness of the underlying rules of pronunciation (Chan and Nunes, 1998). Indeed, those who are good at Pinyin are better readers than those who are poor at using the pronunciation system (e.g., Hu and Catts, 1998), and the association between a word and its pronunciation becomes so strong for skilled readers that the phonological processing of a word is through rapid activation of the representation as a whole (Chua, 1999).

The change of phonological effect from facilitation into inhibition can also be accounted for with reference to the IAM, given that activations of orthographic units simultaneously spread to phonological units (Ferrand and Grainger, 1992). Similar to information processing of orthographic units, phonological units can be processed through excitatory connection between phoneme and lexical levels. At the same time, within-level inhibition from competitive neighbors sharing the same phoneme is also developed.

Instead of being mapped to a phoneme, a Chinese character is generally represented as a single syllable (Li et al., 2013). However, the IAM doesn't postulate syllable-based activation for lexical access, which is otherwise believed to play a crucial role in word recognition (Dominguez et al., 1997). Thus, to explain the current finding, we assume that the syllabic (or phonological) configuration is included in the IAM as a phonological unit that is encoded to activate candidates, just as how the syllable effect was explained in Spanish (Carreiras et al., 1993), German (Conrad et al., 2009), and French (Mathey et al., 2006).

Although the primes were not completely identified yet at the SOA of 47 ms, “information about the logograph's orthographic configuration carried by the early-arriving low spatial frequency components allowed the computation of a ‘prelexical’ phonological code” (Chua, 1999, p. 876). Thus, when a prime (e.g., 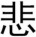) appeared, its phonological information was extracted. The pre-activation of representation for the target (e.g., 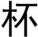), which was the prime's homophone, initiated activation of its semantic representation, facilitating the participants' semantic decision as a result. At the same time, however, the activated phonological representation was to arouse representations for other items (e.g., 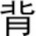 [bei1] *bear*, 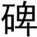 [bei1] *stele*, 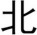 [bei3] *north*, and 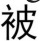 [bei4] *quilt*) that were similar to the prime in pronunciation. When a sufficient level of activation was reached for the prime's representation, activations of representations for those of the phonological competitors, including the target, were inhibited. And in the case of a complete overlap in pronunciation between the candidates activated by the prime and the target, those candidates including the prime would be strongly reactivated during target processing and would act as strong competitors, thereby slowing down the participants' semantic decision on the target.

As with the orthographic inhibition, the phonological inhibition in a priming task increased with time accumulation (Ferrand and Grainger, 1992), thus the competition between the inter-level facilitation and the intra-level inhibition ended up with the former prevailing at the SOA of 47 ms, the latter overweighing at the SOA of 187 ms and the two counteracting at the SOA of 87 ms.

This inhibitory effect for the phonological primes is consistent with Dominguez et al.'s (1997) finding that in an exposure duration of 200 ms, negative priming effect was produced in a lexical decision task when two consecutive words belonging to the same syllabic cohort were processed. Dominguez et al.'s (1997) finding together with the result in Experiment B seems to stand in contrast with the result that a phonological facilitation was observed at longer SOAs (85 and 115 ms) in a naming task (Perfetti and Tan, 1998). This is because a naming task may direct participants' attention to phonological information and lead to an articulatory contamination. Actually, Perfetti and Tan (1998) themselves have also put it that as phonological information in a naming task is more useful than in a non-naming task, the time course results should not be generalized to other tasks.

One may wonder why phonological priming was absent in some previous studies on two-character words (Zhou et al., 1999; Zhou and Marslen-Wilson, 2000, 2009; Wong et al., 2014) but was observed at the SOA of 47 ms in the present experiment. The reason probably lies in methodology. In the present experiment the phonological prime (e.g., 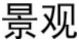 [jing3guan1]) was identical to the target (e.g., 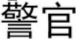 [jing3guan1]) in pronunciation. In Wong et al. (2014) and Zhou et al. (1999), however, the primes were only partially similar to the targets in pronunciation; in Zhou and Marslen-Wilson (2009) the primes that were supposed to be pronounced in the same way as the targets were non-words. Although Zhou and Marslen-Wilson (2000) failed to reveal a significant effect of phonological priming, they did obtain a phonological facilitation of 11 ms in a lexical decision task with an SOA of 57 ms, because they used word primes (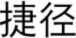 [jie2jing4]) that were identical to the word targets (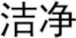 [jie2jing4]) in pronunciation. They would probably have observed a significant priming effect if they had adopted an SOA of 47 ms or shorter. Similarly, one cause for the discrepancy between the phonological priming effects in single characters and two-character words in the literature was because of different degrees of phonological similarity between the primes and the targets. In studies on single characters the primes and the targets were more similar in pronunciation than in those on two-character words in the literature.

In summary, the data in Experiment B suggest that early activations of phonological representations play a role in retrieving the meanings of one- and two-character words. The time-course results further indicate that as with the case of orthographic processing, phonological competition is also involved in word identification.

## General discussion

In the two experiments, we compared the time course of the processing of visual-orthographic, phonological, and semantic information of Chinese words in a forward-masked PTSC (PTSC). The variables were length (one- or two-character words), relation, prime (related or unrelated prime-target pairs), and SOA (47, 87, or 187 ms). The prime was similar to the target in meaning or in visual configuration in Experiment A and in meaning or in pronunciation in Experiment B. In general, we achieved three results. (1) Compared to the control conditions, the semantic priming effect consistently became stronger as SOA increased across the two experiments, confirming the prediction of the SAM. (2) The visual-orthographic primes had an inhibitory effect at the longest SOA in Experiment A; the phonological primes had a priming effect at the shortest SOA and an inhibitory effect at the longest SOA in Experiment B. The inhibition of the visual-orthographic primes and the priming and inhibitory effects of the phonological primes can be explained with reference to the IAM by assuming a word's visual and phonological configurations to be among the subunits. (3) The time-course pattern of the processing of the phonological, visual-orthographic, and semantic information was independent of the stimuli's word length, but the participants had shorter reaction times and lower error rates in responding to the two- than to the one-character targets.

At the SOA of 47 ms, visual-orthographic facilitation was not observed in Experiment A, but phonological priming was revealed in Experiment B. There seem to be two reasons for these different patterns of priming effect across the two experiments. First, while the visual-orthographic primes were just partially overlapped with the targets in visual configurations in Experiment A, the phonological primes were identical to the targets in pronunciations in Experiment B. Compared with the case of the phonological primes in Experiment B, partial overlaps of visual-orthographic information between the visual-orthographic primes and the targets in Experiment A might have resulted in a priming effect that was not strong enough to be observable. Second, in Ferrand and Grainger (1993), a robust orthographic facilitation was observed at the SOA of 17 ms but phonological facilitations only started to emerge at the SOA of 50 ms. Similarly, the SOA of 47 ms might be too long for the priming effect to be observed for the visual-orthographic primes in Experiment A but long enough for phonological facilitation to be reflected in Experiment B.

At the SOA of 87 ms in Experiment B, the increased phonological inhibition counteracted the facilitory effects of phonological priming. At the SOA of 187 ms, inhibitions from both the visual-orthographic and the phonological primes were prominent. The semantic priming, however, became significant at the SOA of 87 ms and was even stronger at the SOA of 187 ms. The delayed availability of the semantic priming compared with that of the phonological primes might be a result of the privileged one-to-one form-form mapping over the one-to-many form-meaning mapping (Perfetti and Tan, 1998).

Given the similarity in the participants' responses to the targets of one and two characters under the influences of relation, prime, and SOA, we argue that the two-character words were taken as wholes in the PTSC. Similar to a one–character word, a two-character word has a definite session of information processing in its visual configuration, phonological configuration, and meaning. Actually, previous studies showed that two-character words can be taken as wholes in lexical decisions (Miwa et al., 2014) and in judgments of semantic relatedness (Liu et al., 2013). Mok (2009) even argued “…all words inevitably develop idiosyncratic functions …The development of whole-word idiosyncratic functions in the course of language usage necessitates a lexical representation for the word as a whole, in order to provide a link for all relevant idiosyncratic information to be activated” (p. 1044).

The result that the two-character words showed an advantage over the one-character words in the participants' performance seems to be at odds with the effect of word length (e.g., Hyönä and Olson, 1995). It is more demanding for cognitive resources to recognize a long than a short word in alphabetic languages. This effect, however, is not always observed (Lovatt et al., 2000). For example, it was found in reading novel words (Lowell and Morris, 2014) or in children's reading (Acha and Perea, 2008) but not in skilled readers' reading of words of high familiarity (Acha and Perea, 2008; Lowell and Morris, 2014). Moreover, consistent with our finding, New et al.'s (2006) simultaneous multiple regression analyses on a selection of 33,006 English words revealed a facilitory effect of word length for words of 3-5 letters. A short word (e.g., *bet*) has more words (e.g., *bit, met, bed*) that are phonetically similar to it than a long word (e.g., *dangerous*) does, and more accumulation of bottom-up evidence is available in recognizing a long than in doing a short word (Pitt and Samuel, 2006). In supporting their argument, Pitt and Samuel (2006) did observe a greater lexical activation for longer than for shorter words in English spoken word recognition.

One reason for the facilitory effect of word length observed in the present study is that one-character words have more ambiguous semantic contents than two-character words (Tan and Perfetti, 1998). Moreover, similar to the fact that long words generally have fewer neighbors than short words (Van Heuven et al., 2001), one-character words have a larger size of phonological and visual-orthographic neighbors than two-character words. Therefore, one-character words are subject to greater inhibitions than two-character words (Pitt and Samuel, 2006).

Actually, only about three and a half thousand basic Chinese characters are required to constitute the more than 70% of the 50,000 most commonly used words (State language affairs committee, 2008). The meanings of many two-character words are related to the meaning of either the constituent characters or to the combined meanings of both constituents (Mok, 2009). In other words, the presentation of a one-character word (e.g., 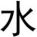 [shui3] *water*) is likely to initiate automatic activations of semantic representations for several two-character words (e.g., 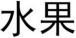 [shui3guo3] *fruit*, 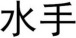 [shui3shou3] *sailor*, and 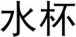 [shui3bei1] *drinking cup*) that contain it as a component character. This argument also is in support of Ji et al.'s (2011) conclusion that availability of lexical and semantic representations for the constituents of a compound helps the compound's recognition.

Considering larger sizes of phonological and visual-orthographic neighbors for one- than for two-character words, one may wonder why interactions were not observed between word length and visual-orthographic or phonological priming in the present study. One explanation is as follows. For both the visual-orthographic and the phonological primes, the inhibitory effects resulted from two stages of information processing: In the first stage, the pre-activations tended to be suppressed for the targets when the primes were being processed; in the second stage, the representations for those that were visual-orthographically or phonologically similar to the targets were likely to be re-activated when the targets were being processed. Having fewer visual-orthographic or phonological neighbors than the one-character targets, the two-character targets were more likely to be pre-activated by the visual-orthographic or the phonological primes than the one-character ones in the first stage. In the second stage, the one-character targets might have received stronger inhibition than the two-character targets because they had a bigger number of re-activated neighbors than the two-character targets.

Skilled readers of Chinese may have developed separate representations for radicals and characters (Ding et al. (2004); the processing of individual characters is inevitable in the recognition of two-character words (Huang et al., 2006). Considering these two points of view and the results of the visual-orthographic primes in Experiment A in combination, we predict that a future study is promising to yield an interesting result in comparing the contributions of a word's visual configuration and its radical or character components in triggering activations of its semantic representation.

## Conclusion

In the PTSC at three SOAs (47, 87, and 187 ms), two-character words are similar to one-character words but are less demanding for cognitive resources than one-character words in the processing of visual-orthographic, phonological, and semantic information. The primes' phonological information played a facilitating role at the SOA of 47 ms but an inhibitory role at the SOA of 187 ms in activating the targets' semantic representations in the present study; the primes' visual configuration played an inhibitory role at the SOA of 187 ms in activating the targets' semantic representations. It is concluded that the visual configuration of a Chinese word of one or two characters has its own contribution in helping retrieve the word's meanings and that the phonological configuration of a one- or two-character word plays its own role in triggering activations of the word's semantic representations.

## Funding

This work was supported by the National Social Science Foundation of China under Grant 11&ZD188, the Social Science Foundation of the Ministry of Education of China under grant 14YJA740016, and the Fundamental Research Funds for the Central Universities.

### Conflict of interest statement

The authors declare that the research was conducted in the absence of any commercial or financial relationships that could be construed as a potential conflict of interest.

## References

[B1] Acha J. Perea M. (2008). The effects of length and transposed−letter similarity in lexical decision: evidence with beginning, intermediate, and adult readers. Br. J. Psychol. 99, 245–264. 10.1348/000712607X224478 17631694

[B2] Carreiras M. Álvarez J. C. de Vega M. (1993). Syllable frequency and orthographic word recognition in Spanish. J. Mem. Lang. 32, 766–780. 10.1006/jmla.1993.1038

[B3] Chan L. Nunes T. (1998). Children's understanding of the formal and functional characteristics of written Chinese. Appl. Psycholinguist. 19, 115–131. 10.1017/S0142716400010614

[B4] Chang L. Y. Xu Y. Perfetti C. A. Zhang J. Chen H. C. (2014). Supporting orthographic learning at the beginning stage of learning to read Chinese as a second language. Int. J. Disabil. Dev. Educ. 61, 288–305. 10.1080/1034912X.2014.934016

[B5] Chen C. Huang X. (1999). Research on characteristics of orthographic recognition to symmetrical structural Chinese characters [In Chinese]. Acta Psychol. Sin. 31, 154–161.

[B6] Chen H. Bukach C. M. Wong A. C. N. (2013). Early electrophysiological basis of experience-associated holistic processing of Chinese characters. PLoS ONE 8:e61221. 10.1371/journal.pone.0061221 23593436 PMC3623809

[B7] Chen H. C. d'Arcais G. B. F. Cheung S. L. (1995). Orthographic and phonological activation in recognizing Chinese characters. Psychol. Res. 58, 144–153. 10.1007/BF00571102

[B8] Chua F. K. (1999). Phonological recoding in Chinese logograph recognition. J. Exp. Psychol. Learn. Mem. Cogn. 25, 876–891. 10.1037/0278-7393.25.4.876

[B9] Collins A. M. Loftus E. F. (1975). A spreading-activation theory of semantic processing. Psychol. Rev. 82, 407 10.1037/0033-295X.82.6.407

[B10] Colombo L. (1986). Activation and inhibition with orthographically similar words. J. Exp. Psychol. Hum. Percept. Perform. 12, 226–234. 10.1037/0096-1523.12.2.226

[B11] Compton A. Bradshaw J. L. (1975). Differential hemispheric mediation of nonverbal orthographic stimuli. J. Exp. Psychol. Hum. Percept. Perform. 1, 246–252. 10.1037/0096-1523.1.3.246 1202147

[B12] Conrad M. Carreiras M. Tamm S. Jacobs A. M. (2009). Syllables and bigrams: orthographic redundancy and syllabic units affect orthographic word recognition at different processing levels. J. Exp. Psychol. Hum. Percept. Perform. 35, 461–479. 10.1037/a0013480 19331501

[B13] Davis C. J. Lupker S. J. (2006). Masked inhibitory priming in English: evidence for lexical inhibition. J. Exp. Psychol. Hum. Percept. Perform. 32, 668. 10.1037/0096-1523.32.3.668 16822131

[B14] Ding G. Peng D. Taft M. (2004). The nature of the mental representation of radicals in Chinese: a priming study. J. Exp. Psychol. Learn. Mem. Cogn. 30, 530–539. 10.1037/0278-7393.30.2.530 14979822

[B15] Dominguez A. de Vega M. Cuetos F. (1997). Lexical inhibition from syllabic units in Spanish orthographic word recognition. Lang. Cogn. Process. 12, 401–422. 10.1080/016909697386790

[B16] Drews E. Zwitserlood P. (1995). Morphological and orthographic similarity in orthographic word recognition. J. Exp. Psychol. Hum. Percept. Perform. 21, 1098–1116. 10.1037/0096-1523.21.5.1098 7595245

[B17] Feldman L. B. Siok W. W. (1999). Semantic radicals contribute to the orthographic identification of Chinese characters. J. Mem. Lang. 40, 559–576. 10.1006/jmla.1998.2629

[B18] Ferrand L. Grainger J. (1992). Phonology and orthography in orthographic word recognition: evidence from masked non-word priming. Q. J. Exp. Psychol. A 45, 353–372. 10.1080/02724989208250619 1308733

[B19] Ferrand L. Grainger J. (1993). The time course of orthographic and phonological code activation in the early phases of orthographic word recognition. Bull. Psychon. Soc. 31, 119–122. 10.3758/BF03334157

[B20] Forster K. I. Forster J. C. (2003). DMDX: a Windows display program with millisecond accuracy. Behav. Res. Methods Instrum. Comput. 35, 116–124. 10.3758/BF03195503 12723786

[B21] Hu C. F. Catts H. W. (1998). The role of phonological processing in early reading ability: what we can learn from Chinese. Sci. Stud. Read. 2, 55–79. 10.1207/s1532799xssr0201_3

[B22] Huang H. W. Lee C. Y. Tsai J. L. Lee C. L. Hung D. L. Tzeng O. J. L. (2006). Orthographic neighborhood effects in reading Chinese two-character words. Neuroreport 17, 1061–1065. 10.1097/01.wnr.0000224761.77206.1d 16791104

[B23] Hyönä J. Olson R. K. (1995). Eye fixation patterns among dyslexic and normal readers: effects of word length and word frequency. J. Exp. Psychol. Learn. Mem. Cogn. 21, 1430–1440. 10.1037/0278-7393.21.6.1430 7490575

[B24] Ji H. Gagné C. L. Spalding T. L. (2011). Benefits and costs of lexical decomposition and semantic integration during the processing of transparent and opaque English compounds. J. Mem. Lang. 65, 406–430. 10.1016/j.jml.2011.07.003

[B25] Leck K. J. Weekes B. S. Chen M. J. (1995). Visual and phonological pathways to the lexicon: evidence from Chinese readers. Mem. Cogn. 23, 468–476. 10.3758/BF03197248 7666760

[B26] Li C. Lin C. Y. Wang M. Jiang N. (2013). The activation of segmental and tonal information in orthographic word recognition. Psychon. Bull. Rev. 20, 773–779. 10.3758/s13423-013-0395-2 23400856

[B27] Liu L. Tao R. Wang W. You W. Peng D. Booth J. B. (2013). Chinese dyslexics show neural differences in morphological processing. Dev. Cogn. Neurosci. 6, 40–50. 10.1016/j.dcn.2013.06.004 23872198 PMC6987802

[B28] Lovatt P. Avons S. E. Masterson J. (2000). The word-length effect and disyllabic words. Q. J. Exp. Psychol. A 53, 1–22. 10.1080/713755877 10718061

[B29] Lowell R. Morris R. K. (2014). Word length effects on novel words: evidence from eye movements. Atten. Percept. Psychophys. 76, 179–189. 10.3758/s13414-013-0556-4 24092359

[B30] Luo Y. C. Chen X. Deacon S. H. Zhang J. Yin L. (2013). The role of visual processing in learning to read Chinese characters. Sci. Stud. Read. 17, 22–40. 10.1080/10888438.2012.689790

[B31] Mathey S. Zagar D. Doignon N. Seigneuric A. (2006). The nature of syllabic neighborhood in French. Acta Psychol. 123, 372–393. 10.1016/j.actpsy.2006.02.003 16620742

[B32] McClelland J. L. Rumelhart D. E. (1981). An interactive activation model of context effects in letter perception: I. An account of basic findings. Psychol. Rev. 88, 375–407. 10.1037/0033-295X.88.5.375 7058229

[B33] Miwa K. Libben G. Dijkstra T. Baayen H. (2014). The time-course of lexical activation in Japanese morphographic word recognition: evidence for a character-driven processing model. Q. J. Exp. Psychol. 67, 79–113. 10.1080/17470218.2013.790910 23713954

[B34] Mok L. W. (2009). Word-superiority effect as a function of semantic transparency of Chinese bimorphemic compound words. Lang. Cogn. Process. 24, 1039–1081. 10.1080/01690960902831195

[B35] Nakayama M. Sears C. R. Lupker S. J. (2008). Masked priming with orthographic neighbors: a test of the lexical competition assumption. J. Exp. Psychol. Hum Percept. Perform. 34, 1236–1260. 10.1037/0096-1523.34.5.1236 18823208

[B36] Neely J. H. (1991). Semantic priming effects in orthographic word recognition: a selective review of current findings and theories, in Basic Processes in Reading: Orthographic Word Recognition, eds Besner D. Humphreys G. W. (Hillsdale, NJ: Erlbaum), 264–336.

[B37] New B. Ferrand L. Pallier C. Brysbaert M. (2006). Reexamining the word length effect in orthographic word recognition: new evidence from the English Lexicon Project. Psychon. Bull. Rev. 13, 45–52. 10.3758/BF03193811 16724767

[B38] Perfetti C. A. Tan L. H. (1998). The time course of graphic, phonological, and semantic activation in Chinese character identification. J. Exp. Psychol. Learn. Mem. Cogn. 24, 101–118. 10.1037/0278-7393.24.1.101 14622057

[B39] Perfetti C. A. Zhang S. (1995). Very early phonological activation in Chinese reading. J. Exp. Psychol. Learn. Mem. Cogn. 21, 24–33. 10.1037/0278-7393.21.1.24

[B40] Pitt M. A. Samuel A. G. (2006). Word length and lexical activation: longer is better. J. Exp. Psychol. Hum. Percept. Perform. 32, 1120–1135. 10.1037/0096-1523.32.5.1120 17002526

[B41] Rumelhart D. E. McClelland J. L. (1982). An interactive activation model of context effects in letter perception: II. The contextual enhancement effect and some tests and extensions of the model. Psychol Rev. 89, 60–94. 10.1037/0033-295X.89.1.60 7058229

[B42] Sakuma N. Sasanuma S. Tatsumi I. F. Masaki S. (1998). Orthography and phonology in reading Japanese kanji words: evidence from the semantic decision task with homophones. Mem. Cogn. 26, 75–87. 10.3758/BF03211371 9519698

[B43] Segui J. Grainger J. (1990). Priming word recognition with orthographic neighbors: effects of relative prime-target frequency. J. Exp. Psychol. Hum. Percept. Perform. 16, 65–76. 10.1037/0096-1523.16.1.65 2137524

[B44] Shen D. Forster K. I. (1999). Masked phonological priming in reading Chinese words depends on the task. Lang. Cogn. Process. 14, 429–459. 10.1080/016909699386149

[B45] Siok W. T. Fletcher P. (2001). The role of phonological awareness and visual-orthographic skills in Chinese reading acquisition. Dev. Psychol. 37, 886. 10.1037/0012-1649.37.6.886 11699761

[B46] State language affairs committee (2008). Lexicon of Common Words in Contemporary China. Beijing: The Commercial Press.

[B47] Taft M. Van Graan F. (1998). Lack of phonological mediation in a semantic categorization task. J. Mem. Lang. 38, 203–224. 10.1006/jmla.1997.2538

[B48] Taft M. Zhu X. (1997). Submorphemic processing in reading Chinese. J. Exp. Psychol. Learn. Mem. Cogn. 23, 761–775. 10.1037/0278-7393.23.3.761

[B49] Tan L. H. Perfetti C. A. (1997). Orthographic Chinese character recognition: does phonological information mediate access to meaning? J. Mem. Lang. 37, 41–57. 10.1006/jmla.1997.2508

[B50] Tan L. H. Perfetti C. A. (1998). Phonological codes as early sources of constraint in Chinese word identification: a review of current discoveries and theoretical accounts, in Cognitive Processing of the Chinese and the Japanese Languages, eds Leong C. K. Tamaoka K. (Dordrecht: Springer), 11–46.

[B51] Tan L. H. Perfetti C. A. (1999). Phonological activation in orthographic identification of Chinese two-character words. J. Exp. Psychol. Learn. Mem. Cogn. 25, 382–393. 10.1037/0278-7393.25.2.382

[B52] Tanenhaus M. K. Flanigan H. P. Seidenberg M. S. (1980). Orthographic and phonological activation in auditory and visual word recognition. Mem. Cogn. 8, 513–520. 10.3758/BF03213770 7219171

[B53] Van Heuven W. J. Dijkstra T. Grainger J. Schriefers H. (2001). Shared neighborhood effects in masked orthographic priming. Psychon. Bull. Rev. 8, 96–101. 10.3758/BF03196144 11340872

[B54] Van Orden G. C. (1987). A ROWS is a ROSE: spelling, sound, and reading. Mem. Cogn. 15, 181–198. 10.3758/BF03197716 3600258

[B55] Wang M. Perfetti C. A. Liu Y. (2003). Alphabetic readers quickly acquire orthographic structure in learning to read Chinese. Sci Stud. Read. 7, 183–208. 10.1207/S1532799XSSR0702_4

[B56] Weekes B. S. Chen M. J. Lin Y. B. (1998). Differential effects of phonological priming on Chinese character recognition. Read. Writ. 10, 201–221. 10.1023/A:1008087715413

[B57] Wong A. W. K. Wu Y. Chen H. C. (2014). Limited role of phonology in reading Chinese two-character compounds: evidence from an ERP study. Neuroscience 256, 342–351. 10.1016/j.neuroscience.2013.10.035 24505608

[B58] Yeh S. L. Li J. L. (2002). Role of structure and component in judgments of orthographic similarity of Chinese characters. J. Exp. Psychol. Hum. Percept. Perform. 28, 933–947. 10.1037/0096-1523.28.4.933 12190259

[B59] Yeh S. L. Li J. L. Takeuchi T. Sun V. Liu W. R. (2003). The role of learning experience on the perceptual organization of Chinese characters. Orthogr. Cogn. 10, 729–764. 10.1080/13506280344000077

[B60] Zhou X. Marslen-Wilson W. (1999). The nature of sublexical processing in reading Chinese characters. J. Exp. Psychol. Learn. Mem. Cogn. 25, 819–837. 10.1037/0278-7393.25.4.819

[B61] Zhou X. Marslen-Wilson W. (2000). The relative time course of semantic and phonological activation in reading Chinese. J. Exp. Psychol. Learn. Mem. Cogn. 26, 1245. 10.1037/0278-7393.26.5.1245 11009256

[B62] Zhou X. Marslen-Wilson W. (2009). Pseudohomophone effects in processing Chinese compound words. Lang. Cogn. Process. 24, 1009–1038. 10.1080/01690960802174514

[B63] Zhou X. Marslen-Wilson W. Taft M. Shu H. (1999). Morphology, orthography, and phonology reading Chinese compound words. Lang. Cogn. Process. 14, 525–565. 10.1080/016909699386185

