# Supplementary material for: The Processing of Visual and Phonological Configurations of Chinese One- and Two-Character Words in a Priming Task of Semantic Categorization
Source: Front Psychol. 2016 Jan 5;6:1918. doi: 10.3389/fpsyg.2015.01918 (PMC4700262; doi:10.3389/fpsyg.2015.01918)
Supplement: Supplementary file 1 [file DataSheet1.docx]

## Appendix A

**The critical materials used in Experiment A**

**
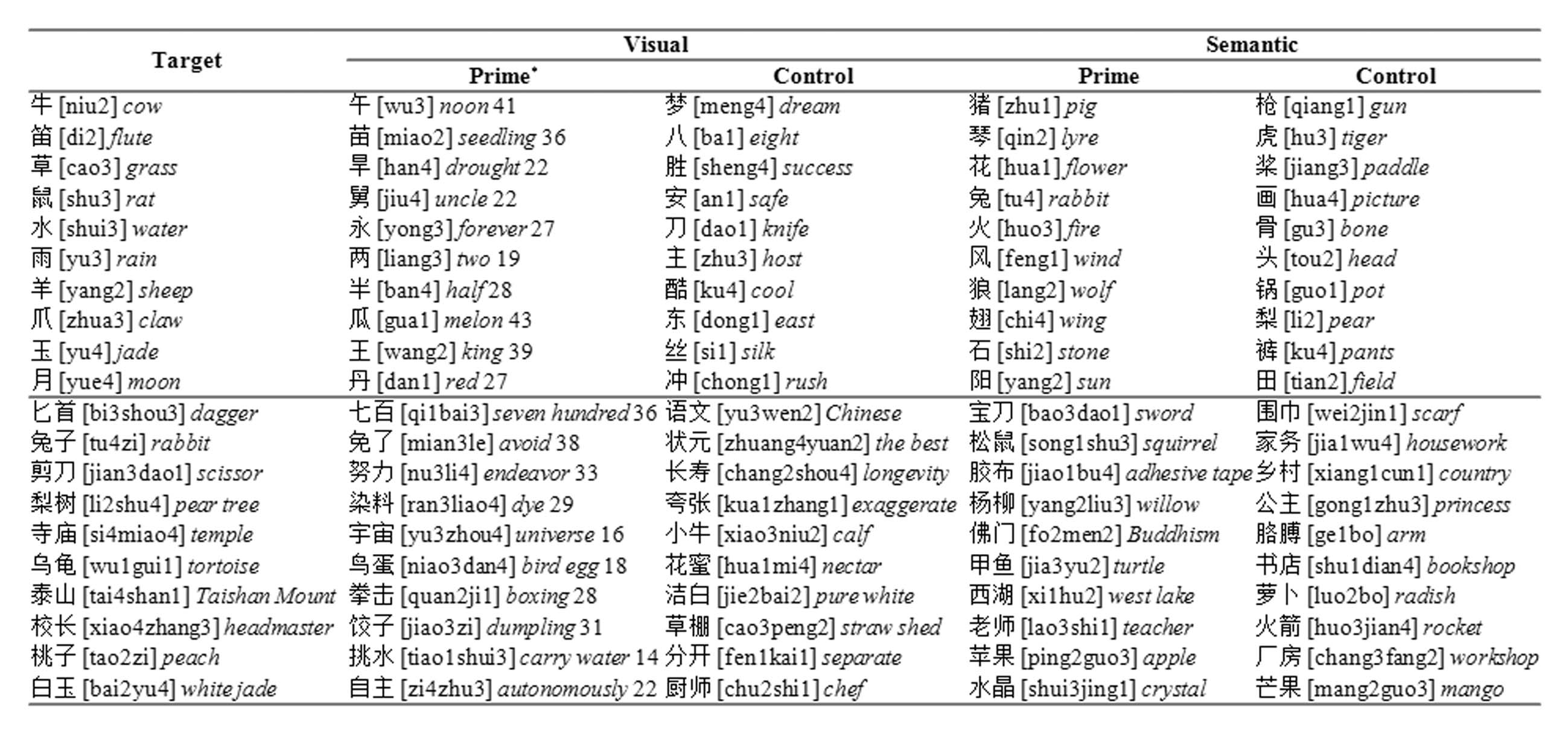
**

** The number after the pronunciation symbol for each item indicates the percentage of participants who provided the visual-orthographic response.*

## Appendix B

**The critical materials used in Experiment B**

*
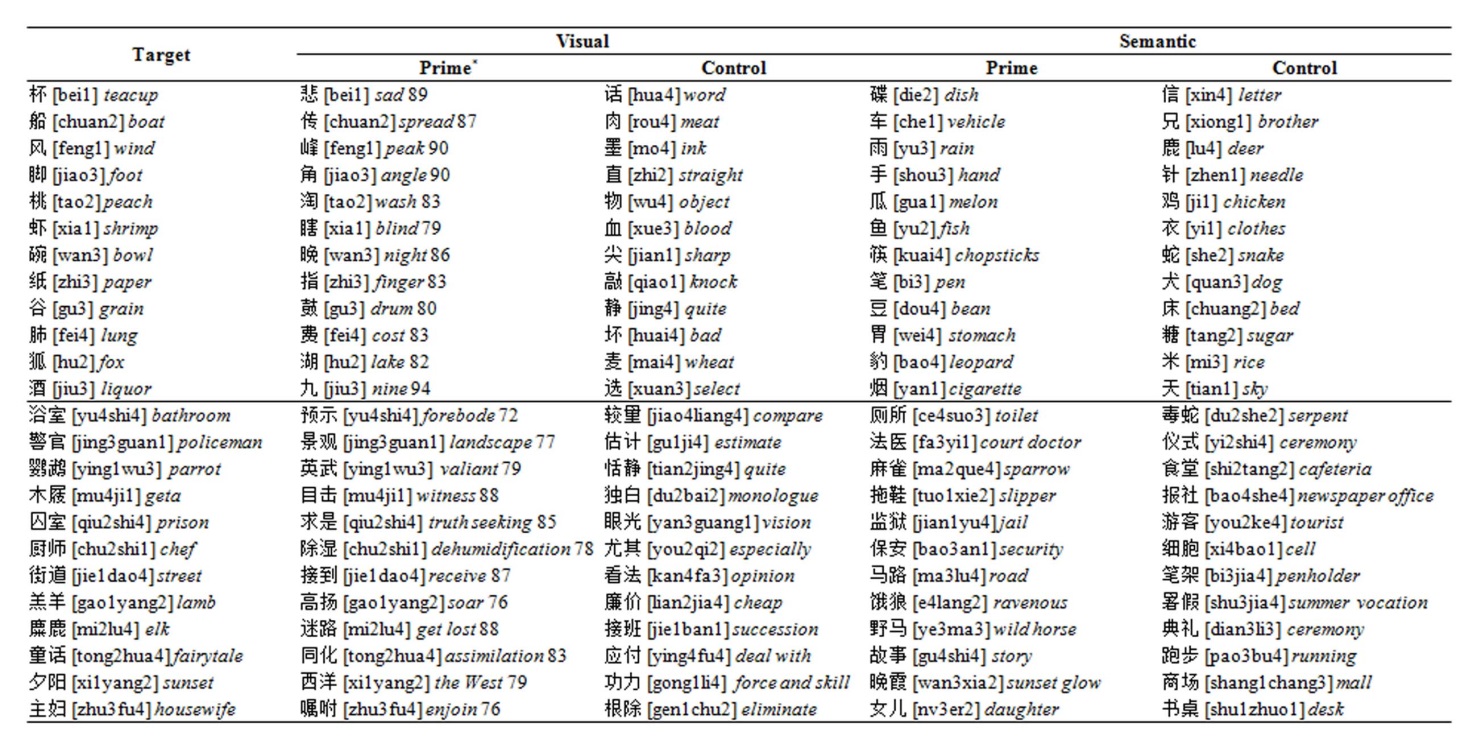
*

** The number after the pronunciation symbol for each item indicates the percentage of participants who provided the phonological response.*
